# Administration of Drugs/Gene Products to the Respiratory System: A Historical Perspective of the Use of Inert Liquids

**DOI:** 10.3389/fphys.2022.871893

**Published:** 2022-05-10

**Authors:** Deepthi Alapati, Thomas H. Shaffer

**Affiliations:** ^1^ Nemours Children’s Health, Wilmington, DE, United States; ^2^ Sidney Kimmel School of Medicine, Thomas Jefferson University, Philadelphia, PA, United States; ^3^ Lewis Katz School of Medicine, Temple University, Philadelphia, PA, United States

**Keywords:** drug delivery, gene delivery, partial liquid ventilation, perfluorochemicals, pulmonary

## Abstract

The present review is a historical perspective of methodology and applications using inert liquids for respiratory support and as a vehicle to deliver biological agents to the respiratory system. As such, the background of using oxygenated inert liquids (considered a drug when used in the lungs) opposed to an oxygen-nitrogen gas mixture for respiratory support is presented. The properties of these inert liquids and the mechanisms of gas exchange and lung function alterations using this technology are described. In addition, published preclinical and clinical trial results are discussed with respect to treatment modalities for respiratory diseases. Finally, this forward-looking review provides a comprehensive overview of potential methods for administration of drugs/gene products to the respiratory system and potential biomedical applications.

## Introduction

Aerosol drugs have been delivered to the lungs for several thousand years (Stein and Thiel, 2017). The use of aerosol delivery is complex, and deposition of drugs in the respiratory system is influenced by several specific factors: physics of the aerosol (inertia of the aerosol), gravitational factors, diffusion (airflow patterns in the lungs), and pulmonary defense mechanisms. Pulmonary drug delivery has been only partly explored in recent decades even though it could represent an alternative route of administration of drug-based therapies. Pulmonary drug delivery is an attractive route of administration of drugs, since the lungs are an ideal entry point for drugs to the bloodstream because of the large surface area, the very short diffusion distances in the alveolar spaces, and exposure to the entire cardiac output. Today there is an increased need for topical delivery of lung cancer therapy drugs, anti-inflammatory drugs to treat acute respiratory distress (i.e., COVID-19, H1N1 influenza), and gene-targeted lung agents for several relatively uncommon (orphan) diseases and pulmonary arterial hypertension (PAH) (Ali et al., 2015; Muralidharan et al., 2015; Alapati et al., 2019; Keshavarz et al., 2020; Kumar et al., 2020).

During the last 20 years, the combination of nanocrystal technology combined with an inert perfluorochemical vehicle has demonstrated the efficacy of large volume drug delivery to the entire lung because of the vehicle physical-chemical properties (inert properties, low surface tension, and high respiratory gas solubility) (Cullen et al., 1999). Furthermore, based on this combination delivery approach, it has been possible to demonstrate increased lung targeted drug delivery as opposed to systemic delivery. Nanocarriers have been found to be most promising because of their significant advantages (i.e., cell-specific targeted drug delivery and prolonged drug release). Thus, in combination with inert perfluorochemical vehicles, nanocarriers may provide effective delivery to the entire lung. The advantages offered by pulmonary drug delivery indicate that the challenges of such a delivery approach are worth addressing; if successfully addressed, there are great opportunities to treat unmet clinical needs. The present review focuses on providing a comprehensive historical perspective of the use of inert liquids for respiratory applications.

## Respiratory Support With Inert Liquids

The use of liquids for respiratory support is reviewed in this section, as well as the physical properties of fluid used and the rationale for using specific liquids.

The first liquid used as a respiratory medium for lung lavage was saline (Winternitz and Smith, 1920). It debrides the lung and eliminates the gas-liquid interface within it. Early saline studies clarified factors influencing distensibility, alveolar structure, stability, pulmonary blood flow, and ventilation (Neergard, 1929; Mead et al., 1957; Avery and Mead, 1959; Leith and Mead, 1966; Hamosh and Luchsinger, 1968; Davidson et al., 1995; Fournier et al., 1995). Low respiratory gas solubility (O_2_; CO_2_) and diffusion gradients at atmospheric conditions limited the functional use of saline solution to provide adequate gas exchange (Kylstra et al., 1966; Kylstra, 1967; Kylstra et al., 1973; Wessler et al., 1977; Lynch et al., 1983). The hypothesis that O_2_-saturated saline solution dissolved under pressure could possibly sustain submersed mammals was formulated (Stein and Sonnenschein, 1950), and subsequent research revealed that adequately oxygenated liquid could be breathed by and support mammals submerged in hyperbaric oxygenated saline solution (Goodlin, 1962; Kylstra et al., 1962; Pegg et al., 1963; Kylstra et al., 1966). However, CO_2_ retention and profound acidosis occurred because of the small gradient between arterial and alveolar CO_2_ gradients, thus eliminating saline ventilation for either normobaric or hyperbaric conditions. In addition, it should be noted that although saline has been used to lavage debris and inflammatory mediators from the lungs as noted above, it has also been shown to inactivate pulmonary surfactant and impair lung function (Shaffer and Wolfson, 2011).

### Inert Perfluorochemical Liquid Physicochemical Properties

As an alternative to saline as a respiratory medium, the utility of other liquids (silicone, vegetable oils, and animal oils) was investigated as respiratory media; however, these oils, although having high gas solubility, also demonstrated toxic effects (Clark, 1970; Sargent and Seffl, 1970). Perfluorochemicals (PFCs) were initially produced as part of the Manhattan Project during World War II. In 1966, they were used to support normobaric respiration on the basis of their high solubility for respiratory gases (Table 1) (Clark and Gollan, 1966), which delineated their use as alternative respiratory mediums. True PFCs are formulated from common organic compounds (e.g., benzene) by substituting carbon-bound hydrogen atoms with fluorine atoms. They provide the advantage of easy storage (indefinitely at room temperature) and can be used under antiseptic conditions without modification (i.e., autoclave, small-pore filtering). They are clear, in most cases, not soluble in aqueous media or nonlipid biologic fluids and are odorless, inert, and transparent—very inoffensive in their use (Shaffer and Wolfson, 2011).

**TABLE 1:** Physiochemical profile of various perfluorocarbons. Reprinted from Shaffer, T.H., and Wolfson, M.R. (2011). “Liquid Ventilation,” in Fetal and Neonatal Physiology, 4th Edition, eds: R. Polin, W.W. Fox, and S. Abman (Philadelphia, PA: WB Saunders), 1063–1081, with permission from Elsevier.

Perfluorocarbon	Formula	Orientation	O_2_ Solution (mL/100 ml) (25 °C)	Vapor Pressure (mm hg) (37 °C)	Boiling Point (°C)	Viscosity (cSt) (25 °C)	Mol wt (g/mol)	Density (g/ml) (25°C)
PP2	C_7_F_14_	Cyclic	57.2	180	76	0.88	350	1.788
PFOB	C_8_F_17_Br	Aliphatic	52.7	11	140.5	1	499	1.89
PCI	C_7_F_15_Cl	Aliphatic	52.7	48.5	108	0.82	404.5	1.77
P12F	C_9_F_20_O	Aliphatic	52.5	39	121	0.95	504.1	1.721
FC-75F	C_8_F_16_O	Cyclic	52.2	51	102	0.85	416.1	1.783
FC-75P	C_8_F_16_O	Cyclic	52.2	51	102	0.85	416.1	1.783
PFDMA	C_12_F_18_	Cyclic	39.4	2.6	177.5	4.35	524.1	2
FC47	C_12_F_27_N	Aliphatic	38.4	2.5	174	2.52	671.1	1.9
PP9	C_11_F_20_	Cyclic	38.4	5.2	160	3.32	512.1	1.972
APF-57	C_6_F_14_	Cyclic	70	356.4	57.3	—	338	1.58
APF-100	C_8_F_16_	Cyclic	42.1	64.6	98.6	1.11	400	1.84
APF-125	C_9_F_18_	Cyclic	47.7	30	116.6	1.17	450	1.86
APF-140	C_10_F_18_	Cyclic	49	13.6	142	2.9	462	1.93
APF-145	C_10_F_20_	Cyclic	45.3	8.9	142.8	1.44	500	1.9
APF-175	C_12_F_22_	Cyclic	35	1.4	180	3.5	562	1.98
APF-200	C_13_F_24_	Cyclic	41	1.26	200	5.3	612	1.99
APF-215	C_14_F_26_	Cyclic	37	0.2	215	8	662	2.02

Please refer to Table 1 (Shaffer and Wolfson, 2011) for details regarding the physicochemical profile and structure of various PFC liquids for PFC ventilation. O_2_ and CO_2_ are specific to respiratory gas exchange and carried only as dissolved gases with solubilities ranging as much as 16 and three times greater, respectively, in PFC than in saline. Oxygen solubilities range from 35 to 70 ml gas per deciliter at 25°C (Riess, 1992). The carrying capacity for CO_2_ is known for only a few PFC compounds, but reported values of CO_2_ solubility are approximately four times greater than those for O_2_ (122–225 ml/dl [i.e., PFOB; perfluorooctylbromide [PFOB] = 225 ml/dl]). It is noteworthy that perflubron (PFOB; perfluorooctylbromide) is the only medical grade perfluorochemical approved by the FDA for emergency medical use. While many properties of PFC liquids vary, they do provide relatively low surface tension and viscosity, and are more dense than both water and soft tissue.

Variations in specific physicochemical properties of the PFC liquids are significant to their use as respiratory media and as vehicles for the administration of biological agents. Fluids of higher vapor pressure may volatilize from the lung more rapidly than liquids having lower vapor pressure. Fluids with greater spreading coefficients (dependent on surface tension) may distribute more easily in the lung than fluids whose spreading coefficients are lower (i.e., FC-75 > PFOB > APF-140) (Weers and Johnson, 1991; Sekins, 1995). Fluids of higher viscosity or kinematic viscosity may balk at redistribution in the lung, thus remaining in contact with a greater area of the alveolar surface for more time than those stratifying with increased rapidity (Miller et al., 1999; Miller et al., 2001) resulting in greater flow resistance.

## Non-Clinical and Clinical Studies With Inert Liquids

The initial preclinical studies in liquid spontaneous breathing and ventilation support were directed at breathing in unusual environments such as deep sea diving, zero gravity, and space travel (Clark and Gollan, 1966; Modell et al., 1973; Moskowitz et al., 1975; Lynch et al., 1983). It was not until the studies with premature lambs (Shaffer et al., 1976; Shaffer et al., 1983a; Shaffer et al., 1983b; Shaffer et al., 1984b; Wolfson et al., 1988) that the application to respiratory distress became evident because of the advantages of low surface tension, improved lung compliance, and gas exchange. As a result of these investigations, the first in extremis FDA-approved total liquid ventilation study in a near-death premature infant with severe respiratory distress was performed (Greenspan et al., 1989). This study and a subsequent study in several critically ill infants (Greenspan et al., 1990) demonstrated that PFC liquid ventilation could support gas exchange and residual improvement in pulmonary function following the return to conventional gas ventilation. The need for a medically approved combination liquid ventilator and medical grade PFC breathing fluid restricted further clinical trials. It is noteworthy, however, that a corporate-sponsored multicenter trial resulted from the success of the neonatal and adult animal trials with PFC liquid-assisted gas ventilation and the initial clinical trials with human subjects.

Subsequently, several separate investigational new drug applications were approved by the FDA to investigate the safety and efficacy of PFCs, mainly PFOB, as a liquid breathing media in neonates. While animal studies over the years showed significant efficacy and safety of liquid breathing, clinical studies using several techniques in humans (infants, children, and adults) had mixed outcomes. The findings from non-clinical and clinical studies are summarized below.

### Non-Clinical Studies With Inert Liquids

Over the course of the last 50 years, many animal studies demonstrated liquid ventilation to be an effective approach/treatment for deep sea diving, zero gravity, severe lung injury, and congenital diaphragmatic hernia (CDH). These studies supported the use of liquid ventilation as a superior source of respiratory support when compared with gas media with spontaneous breathing or conventional mechanical ventilation (CMV). Various studies also demonstrated short-term beneficial physiologic responses in lung function because of improved alveolar recruitment and significant preservation of normal histological structure of the lung (Moskowitz et al., 1975; Shaffer et al., 1983a; Shaffer et al., 1983b; Shaffer et al., 1984a; Shaffer et al., 1984b; Wolfson et al., 1992; Leach et al., 1993; Richman et al., 1993; Sekins et al., 1994; Major et al., 1995; Al-Rahmani et al., 2000; Cox et al., 2003). Non-clinical studies in newborn animal models of respiratory distress syndrome (RDS) showed that PFOB enhances uniformity of the lung inflation consistent with PFOB working as an artificial surfactant (Weis et al., 1997; Wolfson et al., 1998; Kandler et al., 2001; Hübler et al., 2002; Merz et al., 2002). Animal studies also showed that PFOB minimizes functional lung impairment because of the high airway pressures and sustained FiO_2_ requirements that are characteristics of ventilator-induced lung injury (Greenspan et al., 1990; Wolfson et al., 1992; Bateman et al., 2001; Davies et al., 2002).

Recent studies continue to show PFOB improves oxygenation (Hartog et al., 1997; Bleyl et al., 1999; Al-Rahmani et al., 2000; Bateman et al., 2001; Davies et al., 2002) in animal models of lung injury consistent with earlier findings. Additionally, recent studies report that PFOB increases lung compliance (Bleyl et al., 1999; Al-Rahmani et al., 2000; Kandler et al., 2001; Davies et al., 2002). Findings from earlier studies indicated that PFOB may have potential anti-inflammatory properties. Animal studies showed that administration of PFOB decreased the expression of known inflammatory markers (Kawamae et al., 2000; Haeberle et al., 2002; Merz et al., 2002). Two additional research studies reported PFOB not interfering with cerebral blood flow (Davies et al., 2010; Davies et al., 2013), suggesting partial liquid ventilation (PLV) with PFOB will have limited impact on cardiac output and circulation.

Animal studies consistently support the safety of PFOB, as few negative effects have been reported. Studies show PFOB is not absorbed systemically and causes no long-term harm (Holaday et al., 1972; Shaffer et al., 1996; Cox et al., 2002). Several animal studies reported final concentrations of PFC measured within the blood and tissue after the end of treatment were considered minimal compared with their baseline control measurements (Holaday et al., 1972; Modell et al., 1973; Shaffer et al., 1996; Cox et al., 2002). Additionally, studies revealed initiation of PLV and removal of PFOB does not produce significant adverse effects. Pneumothorax, a documented adverse event in adult PFOB clinical studies, was not reported with any significance in animal models of severe lung injury employing PFOB.

### Clinical Studies in Infants With Inert Liquids

Early clinical studies demonstrated that infants with severe RDS, meconium aspiration, and CDH tolerated liquid PFC in their lungs and were able to effectively exchange gas and maintain cardiovascular stability (Greenspan et al., 1989; Greenspan et al., 1990; Gross et al., 1995; Pranikoff et al., 1996; Henrichsen et al., 2012).

Consistent with those studies and animal studies, additional published reports indicated PFOB increased gas exchange (Hirschl et al., 1995; Gauger et al., 1996; Hirschl et al., 1996; Hirschl et al., 2002) in adults and children. Subsequent studies using PFOB on preterm neonates also reported increases in lung compliance (Greenspan et al., 1990; Hirschl et al., 1995; Hirschl et al., 1996). PFOB rapidly improved lung function and increased survival in a population of neonates with high mortality (Leach et al., 1996).

Most importantly, the utility of PFOB was demonstrated in a multicenter study of premature infants with severe RDS refractory to other available treatments (surfactant therapy, high frequency, too young for extracorporeal membrane oxygenation) (Leach et al., 1996). Thirteen (13) infants were treated with PFOB. Within an hour following the instillation of PFOB, there was an increase in arterial oxygen tension of 138%. Dynamic compliance increased by 61% and continued to climb through the first 24 h (Leach et al., 1996). Furthermore, the mean oxygenation index, markedly elevated at baseline (49 ± 60), fell to 17 ± 16 within the first hour and continued to fall to 9 ± 7 at 24 h. Arterial carbon dioxide tension normalized within 4 h after PFOB treatment. Mean airway pressure decreased from 17 ± 3 to 12 ± 2 cm of water (29%) in the first 24 h despite an increase in tidal volume (5.0 ± 3.4 ml/kg during gas ventilation to 7.8 ± 3.4 ml/kg during PFOB ventilation). It should be noted that no serious adverse events were reported during PFOB-assisted ventilation. It was determined that PFOB-assisted ventilation could be utilized for critically ill infants for several days without serious adverse events. A number of the surviving participants are currently well and in their twenties.

More recently, lavage with PFC has been shown to be safe for treatment of persistent and difficult-to-treat lung atelectasis (Henrichsen et al., 2012). Bronchoalveolar lavage utilizing PFC liquid was performed without incident in infants with severe alveolar proteinosis during conventional mechanical ventilation without necessitating the additional support of extracorporeal membrane oxygenation. Furthermore, recent PFC liquid studies have reported safe imaging studies in bronchopulmonary patients (Degnan et al., 2019).

Follow-up imaging studies up to 20 years after treatment with PFOB in humans demonstrated no negative effects from this treatment (Tiruvoipati et al., 2007; Hagerty et al., 2008; Servaes and Epelman, 2009). The studies demonstrated evidence of residual PFC specs in the lung, thorax, mediastinum, and retroperitoneum. These are also cautionary when interpreting the high-density opacifications associated with Hounsfield unit densities of some PFCs used with intrapulmonary applications such as pulmonary calcification (Tiruvoipati et al., 2007) and stress the necessity of obtaining precise clinical histories in the light of unusual radiographic findings (Hagerty et al., 2008).

### Clinical Studies in Adults With Inert Liquids

In early liquid ventilation studies, it was reported that PFOB usage in adults increased lung compliance (Hirschl et al., 1996), which is consistent with preclinical animal studies. However, a randomized clinical trial in adults with acute respiratory distress syndrome (ARDS) randomized to protective conventional mechanical ventilation of the lung, low-dose PFC, or high-dose PFC and partial liquid ventilation did not result in improved mortality. Additional ventilator-free days were realized in the conventional mechanical ventilation group when compared with the low-dose and high-dose PFC groups (Hirschl et al., 2002; Degraeuwe and Zimmermann, 2006; Kacmarek et al., 2006). Improved mortality or ventilator-free days did not result in another randomized clinical trial in spite of decreased progression in respiratory insufficiency to ARDS in patients treated with partial liquid ventilation with PFC.

## Drug/Gene Product Administration

Systemic administration of therapeutics to target the lung is faced with numerous challenges secondary to potential degradation by serum and hepatic enzymes and rapid renal clearance. Compromised pulmonary blood flow in the injured lung may further limit passive diffusion of the drug from the blood into the lung parenchyma. Retention of the therapeutics in the lung is also often suboptimal. These challenges can be mitigated by local administration of therapeutics through inhalation or airway instillation (Bennett et al., 2002). However, many acute and chronic lung diseases affect distally located alveoli and thus require delivery of biological agents to the distal lung parenchyma for optimal therapeutic effect. Distal delivery of therapeutic agents requires obtaining the correct particle size, which has been challenging. Furthermore, ventilation abnormalities in the impaired lung regions also minimize drug delivery to these target areas.

### Drug Delivery With Inert Liquids

Some studies have successfully demonstrated that lungs filled with PFC liquid have the ability to deliver active and inactive agents for the diagnosis and treatment of respiratory disorders.

Respiratory infections affect distally located alveoli with bacteria and viruses multiplying in the alveolar cells. Distal lung distribution of intra-tracheally delivered anti-infective agents is essential to halt disease progression. In a newborn lamb model of acid lung injury, gentamicin administration during tidal liquid ventilation using PFC resulted in significantly higher lung gentamicin levels compared with intravenous administration (Fox et al., 1997; Zelinka et al., 1997; Cullen et al., 1999; Cox et al., 2001). This technique also resulted in greater pulmonary gentamicin levels and lower serum levels, which are required to achieve therapeutic levels in the lung while mitigating systemic adverse effects of gentamicin. Similarly, when utilizing a piglet model of meconium aspiration, greater pulmonary levels and lower serum levels of vancomycin were achieved after intrapulmonary instillation of vancomycin and PFC followed by partial liquid ventilation, compared with intravenous injection of vancomycin followed by conventional gas ventilation (Jeng et al., 2007).

In an animal model of meconium aspiration syndrome, partial liquid ventilation improved regional distribution of intratracheally administered radio-labelled surfactant compared with conventional mechanical ventilation. There was more uniform distribution of surfactant between the lungs as well as between both ventral and dorsal regions of the lungs. Such uniform distribution was associated with improved systemic oxygenation (Chappell et al., 2001). PFC was also an effective delivery vehicle for pulmonary administration of vasoactive agents (Wolfson et al., 1996).

Inhalational smoke-induced acute lung injury is characterized by airway epithelial injury leading to excess leakage of plasma substrates into large airways and the formation of fibrin casts. Interventions to prevent or treat airway casts are limited. In this regard, PFC has been used for intratracheal administration of plasminogen activators (tissue plasminogen activator [tPA] and single-chain urokinase plasminogen activator [scuPA]) for management of airway clot and fibrinous cast formation associated with smoke-induced acute lung injury. Enzymatic activities of the plasminogen activator following dispersion and storage in PFC were preserved, and PFC administration alone did not impact physiologic or histological differences. In contrast, PFC-facilitated plasminogen activator delivery resulted in significantly better physiologic and histologic outcomes. PFC-facilitated delivery of plasminogen activator demonstrated improved outcomes than achieved by nebulization of plasminogen activators alone (Wolfson et al., 2020).

Drug delivery during PFC PLV respiratory support has been demonstrated with other soluble gases in PFOB such as inspired nitric oxide (NO). NO administration with PLV in surfactant-depleted adult pigs resulted in a significant improvement in gas exchange and decrease in pulmonary artery pressure, most notably without deleterious effects on systemic hemodynamic conditions (Houmes et al., 1997). In a congenital diaphragmatic hernia lamb preparation treated prophylactically with PLV, it was demonstrated that NO improved oxygenation and reduced pulmonary hypertension (Wilcox et al., 1994). As such, the ability to deliver NO during PLV is probably related to distribution of NO in the gas-ventilated regions of the lung, the solubility and diffusion of this gas in the PFC, and recruitment of lung volume. Results on the effective delivery of NO in PFC liquid are consistent with earlier studies showing the use of PFC liquid as a vehicle to deliver biologic agents. Based on transport principles, it appears that the amount of NO delivered to pulmonary structures is dependent on NO concentration in PFC liquid, stratification pattern of gas and PFC liquid in the lung, distribution of pulmonary blood flow, and ventilation-perfusion matching. Finally, the clearance of NO from the partially filled PFC lung and potential formation of NO_2_ during liquid ventilation potentially could be different compared with the gas-filled lung.

### Gene Delivery to the Respiratory System

Twenty-two percent of all pediatric hospital admissions are due to respiratory illness. Genetic lung diseases account for increased morbidity and mortality (Nogee, 2010; Tanash et al., 2010; Witt et al., 2012). Genetic diseases such as surfactant protein disease, cystic fibrosis, Hermansky-Pudlak syndrome, and neuroendocrine cell hyperplasia of infancy (NEHI) cause severe lung disease and are associated with high mortality and morbidity. No cure currently exists. Pulmonary epithelial cell-specific genetic mutations and abnormal gene regulation play a causal role in many genetic lung diseases and are attractive targets for airway delivery of therapeutic agents. Effective airway-based delivery of gene therapy vectors is a substantial hurdle to successful gene therapy for lung diseases.

CRISPR-Cas9 gene editing provides an unprecedented opportunity to manipulate genes in somatic cells. Editing technologies have demonstrated clear therapeutic promise in non-human primates and early human clinical trials (Komor et al., 2016; Maeder et al., 2019; Frangoul et al., 2021; Musunuru et al., 2021; Rothgangl et al., 2021). New approaches in base editor design enable installation of targeted, single-nucleotide mutations without double strand breaks or the need for donor DNA templates, an exciting advance that paves the way for correction of single nucleotide polymorphisms that comprise the largest class of known pathogenic genetic variants in humans (Landrum et al., 2016; Stenson et al., 2017). Genetic surfactant protein diseases are a particularly attractive target given that the lung is a barrier organ amenable to intratracheal or nasal treatment applications to selectively reach pulmonary cell lineages (Alton et al., 2015; Alapati et al., 2019; Kang et al., 2020).

The postnatal lung presents important limitations to airway delivery because of the substantial mucus and surfactant barrier at the air-epithelial interface, repulsive charge interactions at the cell membrane, and unequal reagent distribution due to the heterogeneity of lung disease with some areas being overinflated and others collapsed (Kim et al., 2016; Alapati and Morrisey, 2017; Roesch and Drumm, 2017). Furthermore, proteinaceous debris and inflammatory fluids contribute to additional physical barriers in diseased lungs. These barriers limit adequate ventilation, particularly to diseased tissues, resulting in limited delivery of inhaled therapies to needed locations. Systemic drug delivery to diseased lung may be affected by the displacement of blood flow away from the injury site. In contrast, any related absence of such immune and physical barriers in the fluid-filled fetal lungs has resulted in systematic gene transfer to the pulmonary epithelial cells following prenatal viral vector delivery by intra-amniotic injection, taking advantage of fetal breathing movements for lung targeting (Buckley et al., 2005; Endo et al., 2010; Joyeux et al., 2014; Alapati et al., 2019). Additionally, the fluid-filled fetal lung supports relatively uniform targeting of most major pulmonary epithelial cell types, including distal and proximal lineages (Alapati et al., 2019).

### Gene Product Delivery With Inert Liquids

The fluid-filled fetal lung physiology could be partially mimicked in postnatal lungs with PFC liquids because of their high spreading coefficients and intriguing properties for pulmonary distribution of biological agents. The high O_2_ and CO_2_ solubility of PFC liquids allows effective gas exchange, even in lungs filled with fluid. PFCs also reduce surface tension, which assists lung volume recruitment at reduced inspiratory pressures by eliminating the air-liquid interface. Importantly, PFC liquids effectively penetrate collapsed regions of the lung to facilitate access to under-ventilated regions. This is particularly important in non-homogenous lung diseases such as surfactant protein diseases, in which PFC liquids could simultaneously facilitate delivery of therapies, increase gas exchange, and improve pulmonary function. Several groups have exhibited the superior effectiveness of PFC liquids as vehicles for pulmonary distribution of genetic cargo (Lisby et al., 1997; Weiss et al., 1999a; Weiss et al., 1999b; Weiss et al., 2000; Kazzaz et al., 2011). PFC liquids instilled during the intratracheal administration of recombinant viral vector propelled the vector more effectively into the lung. As a result, PFC enhanced airway and alveolar epithelial gene expression in both normal and injured rodent lungs (Weiss et al., 2001). Use of PFC as vehicle for delivery of genetic cargo also resulted in earlier detection of gene expression and need for lesser amounts of vector. In all of these studies, PFC was administered immediately following administration of aqueous vector because of immiscibility of PFC with aqueous solutions. The propulsive effect of PFC resulted in improved delivery and distribution of the vectors. PFC liquids also transiently decreased transepithelial resistance and increased tight junction permeability. This transient increase in epithelial permeability enhanced access to viral vectors and gene expression. The peak effect was observed from 6 h to 1 day following instillation. Notably, alveolar-capillary permeability was not affected (Weiss et al., 2003). Many studies have also demonstrated improvement in lung mechanics and oxygenation in research models of lung injury following administration of PFC liquids in nebulized or aerosolized forms (Bleyl et al., 1999; Ragaller et al., 2001; Kandler et al., 2004; von der Hardt et al., 2004). As such, administration of nebulized perflubron improved resulting recombinant viral vector mediated gene expression (Beckett et al., 2012). By adapting and optimizing PFC liquid strategies demonstrated to be beneficial for viral vector gene delivery, PFC liquids hold promise for enhanced airway delivery of CRISPR systems as therapeutic strategy for a myriad of respiratory disorders.

### Considerations for Improving Drug/Gene Delivery With Inert Liquids

The fact that aqueous solutions are not readily soluble in PFCs is an important consideration for PFC drug delivery. Some research projects have circumvented this barrier by relying on bulk flow turbulent mixing (Wolfson et al., 1996; Lisby et al., 1997). However, techniques that improve solubility of the drug or biologic agent in PFC are advantageous to provide stability and equivalent disbursement of the drug within the lung and for more controlled dosing procedures. One such method is generation of nanocrystals that can then be administered during partial liquid ventilation. This approach was successfully utilized by developing gentamicin/perfluorochemical nanocrystal suspension that was delivered using two techniques (Cullen et al., 1999). In the first technique, called the top-fill technique, gentamicin/PFC nanocrystal suspension was instilled through the sideport of an endotracheal tube 29 ± 8 min after initiation of partial liquid ventilation with a bolus of oxygenated perflubron. In the second technique, called the slow-fill technique, the gentamicin/PFC nanocrystal suspension was combined with perflubron, vortexed, and delivered through the sideport of an endotracheal tube. Thus, in the second technique, partial liquid ventilation and gentamicin treatment were initiated simultaneously. Both the techniques resulted in effective distribution of gentamicin into the lung and greater gentamicin levels per gram of dry lung tissue compared with intravenous administration of aqueous gentamicin. The amount of original gentamicin dose left in the lobes of the lungs adjusted for dry weight after 4 h and was greater in the slow-fill technique compared with the top-fill technique.

## Summary

When lung parenchymal disease and/or injury are present in the lung, pulmonary ventilation and perfusion are compromised. Ventilation can be irregular and perfusion may be inhibited by ventilation-perfusion mismatch. The route of therapy administration is hindered by these abnormalities, rendering standard intravenous and aerosol/endotracheal tube delivery ineffective in delivering therapeutic agents to the affected area.

Liquid ventilation with an inert respiratory gas solubility is a revolutionary mode for respiratory support, as well as delivery of drug/gene product to the respiratory system. As noted, inert perfluorochemical liquids have low viscosity and high oxygen and carbon dioxide capabilities (Grotberg, 2001). The physical properties of PFC liquids improve lung mechanics and gas exchange and condition the lung parenchymal surface for optimal administration of drug/gene product.

The use of PFC liquid in the respiratory system enhances ventilation and perfusion matching, boosting exposure of the drug/gene product to the circulation, successfully reaching required therapeutic serum drug levels (Fox et al., 1997). Studies have demonstrated the utilization of PFCs as adjuncts for intrapulmonary biological agent delivery both preclinically and clinically as reported herein.
